# The Influence of Multiculturalism and Assimilation on Work-Related Outcomes: Differences Between Ethnic Minority and Majority Groups of Workers

**DOI:** 10.5334/pb.472

**Published:** 2019-07-16

**Authors:** Patrizia Villotti, Florence Stinglhamber, Donatienne Desmette

**Affiliations:** 1 Département d’éducation et pédagogie, Université du Québec à Montréal, Montréal, CA; 2 Centre de recherche de l’Institut Universitaire en Santé Mentale de Montréal, Montréal, CA; 3 Institut de recherche en sciences psychologiques, Université catholique de Louvain, Louvain-la-Neuve, BE

**Keywords:** multiculturalism, assimilation, dual identity, job satisfaction, intention to quit, minority

## Abstract

This study aims at acquiring knowledge on how to manage ethnic diversity at work in order to promote work-outcomes in minority and majority groups of workers. We tested a model on how assimilation and multiculturalism, endorsed at an organizational level, predict job satisfaction and intention to quit through a mediation role played by the identification of workers with both the organization and their ethnic group simultaneously (i.e., dual identity). We hypothesized that the indirect effects of multiculturalism on work outcomes via dual identity are stronger for minority and those of assimilation are stronger for majority. Data came from 261 employees who responded to an online survey. 77 were of foreign origin (minority group) and 184 were of Belgian origin (majority group). Both assimilation and multiculturalism relate positively to work-related outcomes for both groups. However, multiculturalism through dual identity has the most beneficial outcomes for workers of the minority group. Our findings highlight the need to take ethnic and identity issues in account when studying work outcomes in culturally diverse organizations.

## Introduction

Changes in the demographic and ethnic composition of the workforce, as well as new legislations worldwide and labor trends, make diversity more and more prevalent in modern workplaces. As a result, employees with different cultural backgrounds, nationalities, skills and experiences come together and interact daily inside organizations. Belgium is a population that boasts diverse cultures and ethnicities, with foreign people reaching the rate of 29.3% of the total population (SPF, 2015).^1^ Most of the foreign population in Belgium has their origin in EU countries (i.e., France, Italy, the Netherlands; Eurostat, 2012) due among others to workers of the European Institutions in Brussels. North- and other regions of Africa are one of the most representative groups (23.7%) of non-EU foreign people (SPF, 2015). Yet, this group has a lower employment rate on the Belgian labor market than the Belgian natives (Eurostat, 2012).

Gaining a better understanding of how to manage ethnic diversity in organizations is of prime importance to prevent discrimination, to promote well-being of minorities in the workplace, and to finally benefit from diversity (e.g., in terms of performance in work groups, Guillaume et al., 2014). Belgium promotes mixed policies of diversity that combine mainly equality of treatment between individuals (e.g., non-discriminatory treatment) and acknowledgment of cultural differences (e.g., accommodations) (Koninklijk Commissariaat voor het Migrantenbeleid, 1989; SPF 2010; Rea, 2014). On this basis, we may consider that both colorblindness and multiculturalism co-exist in the Belgian context. Colorblindness refers to the belief that people should be judged as individuals without regard to race or ethnicity while multiculturalism refers to the belief that differences among ethnic groups should be recognized and embraced (Rosenthal & Levy, 2012). However, research showed that concerns about the feasibility of colorblind exist and that assimilation, that is the full adaptation to the mainstream culture without maintaining one’s original minority culture (Berry, 2001), may prevail when people ignore group distinctions (Rosenthal & Levy, 2010).

In line with this view, several researchers (Lu, Samaratunge, & Charmine, 2013; Olsen & Martins, 2016) examined the effects of diversity in the frame of acculturation (Berry, 2001). Organizational acculturation refers to “the way in which an organization deals with the existence of multiple social or cultural groups in its workforce” (Olsen & Martins, 2016, p. 4). Within the diversity literature, assimilation and multiculturalism have mainly been analysed as different and competing interethnic ideologies for how an ethnically diverse group should optimally be integrated (see for example Rosenthal & Levy, 2010; Oerlemans & Peeters, 2009). Also, in the specific literature on cultural diversity at work, the conditions under which ethnic differences enhance or detract from work group functioning are described on the one hand as a resource for learning and adaptive change, and on the other hand as a basis for discrimination and conflict, for which it would be preferable to assimilate to the dominant culture (see Ely & Thomas, 2001).”

Diversity in organizations has been reported as leading to mixed (i.e., positive and negative) outcomes (see William & O’Reilly, 1998 for a review). In this study, we focused on assimilation and multiculturalism and two outcome variables, i.e. job satisfaction and intention to quit. Generally, when individuals can display their cultural heritage, their satisfaction and commitment increase: employees report to be more satisfied (Verkuyten et al., 1993) and committed (Van der Zee et al., 2004) in their job when they frequently work together with ethnically similar colleagues. However, this effect is stronger for the members of the minority group (Hofhuis et al., 2012). Moreover, the literature has shown that benefits of diversity are much greater in multicultural societies and organizations (Ely & Thomas, 2001; Rosenthal & Levy, 2010). This has been explained by the fact that multiculturalism values positive mutual intergroup differentiation (Hewston & Brown, 1986) and that within multicultural contexts, people affiliate to multiple social groups and develop *dual identities* (Iweins, Desmette, Yzerbyt, & Stinglhamber, 2013; Goclowska & Crisp, 2014). Dual identity is the combination of both group identity and a superordinate identity, such as organizational identity (Dovidio et al., 2007). In line with the literature on the concept of social identity complexity (Roccas & Brewer, 2002; Brewer, 2010), social identities can be combined following different patterns, including the one of dominance, where the individuals adopt one primary group identification to which all the other potential group identities are subordinated, and the compartmentalization one, in which social identities are context specific (for example, the work environment). In the workplace, employees might define themselves in terms of organizational identities, especially when they are proud of the organization (normative fit; Haslam, Postmes, and Ellemers, 2003). Yet, other forms of social identity are contextually possible, such as ethnicity. The construct of dual identity can be central in reducing tensions between majority and minority groups (Hofhuis et al., 2012), because the perception of differences between the groups at one level (i.e., group identity) is weakened by a countervailing accentuation of perceived similarities at the other level (that is the shared level, i.e., the common identity) (Hornsey & Hogg, 2000). However, how the process of dual identity performs within culturally diverse work teams is still to be understood.

In general, current research in this domain presents several limitations. First, as Ashikali and Groenveld (2015) noted, diversity management is frequently criticized for being primarily supportive for the position of minority groups without considering that acculturation is supposed to concern both minorities and majorities (Berry, 2001; Olsen & Martins, 2016). Second, many studies on how organizations manage diversity measure multiculturalism only, without considering assimilation (e.g., Ashikali & Groeneveld, 2015; Triana et al., 2009). Yet, in some contexts like in Belgium, both assimilation and multiculturalism are simultaneously present and promoted (Koninklijk Commissariaat voor het Migrantenbeleid, 1989; Rea, 2014). Third, the processes through which diversity perspectives enhance organizational outcomes and contribute to workers’ career is still largely unknown (Akkermans & Kubbasch, 2017; Hofhius et al., 2016). In that respect, the role of identity processes in diversity contexts remains not fully understood (Shore et al., 2011), particularly in relation to work identity (Akkermans & Kubbasch, 2017; Olsen & Martins, 2016). In fact, both group and work identities (i.e., dual identity) might be strengthened in assimilationist and in multicultural contexts, depending on which group is considered.

Filling these gaps, our paper contributes to the literature by examining how both organizational multiculturalism and organizational assimilation influence work-related outcomes (i.e., job satisfaction and intention to quit) through dual identity among the minority and the majority group of workers. In this study, dual identity refers to the simultaneous presence of both the identity related to origin and the organizational identity. In the next section of the paper, we present the theoretical background leading to the development of our hypotheses.

### Multiculturalism and assimilation

The debate regarding which diversity perspective (i.e., assimilation, multiculturalism) leads to greater gains is still very vivid in the scientific field (Arasaratnam, 2013; Rosenthal & Levy, 2010). Most of the studies focus on antecedents (e.g., Verkuyten & Wolf, 2002), rather than outcomes, of adhering to one or the other diversity perspective, and in general both multiculturalism and assimilation have been studied mainly from the individual point of view (see for example Oerlemans & Peeters, 2009; Rattan & Ambady, 2013; Verkuyten, 2005). Studies on intergroup attitudes as outcomes of adhering to multiculturalism or assimilation show an association between multiculturalism and more positive intergroup relations, such as less discrimination (Brolis, et al., 2018; Richerson & Nussbaum, 2004), inclusive attitudes (Wolsko et al., 2006) and greater acceptance of and openness to others (Verkuyten, 2005; Vorauer et al., 2009). Large amounts of research also show that multiculturalism is the most adaptive acculturation strategy for migrants (i.e., workers who are perceived as disadvantaged regarding their originated country and ethnic origin, Al Ariss & Crowley-Henry, 2015) to integrate themselves in a new society (reviews of Nguyen & Benet-Martines, 2012; Sam & Berry, 2006). From the perspective of majority groups though, multiculturalism can be perceived as a threat to the national identity when it is embraced at a concrete, rather than abstract manner (Mahfud, Badea, Verkuyten, & Reynolds, 2018; Yogeeswaran & Dasgupta, 2014). Presenting multiculturalism highlighting its broad goals rather than concretely determine the manners with which to achieve them, seems the key for positive majority groups’ attitudes toward ethnic minorities. Multiculturalism can be defined as an ideology that acknowledges and celebrates cultural differences (Arasaratnam, 2013; Wolsko, Park & Judd, 2006). It therefore recognizes diversity, promotes and encourages the distinctiveness of ethnic minorities, and defends an ideal of distinct cultural communities living side-by-side following the principle of equal value of cultures (Kelly, 2005).

In contrast with multiculturalism, assimilation emphasizes that people are basically the same so that ethnic differences should be ignored and conformed into the mainstream culture (Berry, 2001; Wolsko et al., 2006). Assimilation is based on the assumption of similarity between minority groups and the adopting society, so that a minority group can be successfully integrated into the mainstream society, and harmonious intergroup relations within the receiving societies can be achieved. Thus, by definition, assimilation assumes a spontaneous absorption of ethnic minorities within the majority’s culture. Based on this assumption, no policy measure should be taken on cultural differences: society is seen as a whole, and group differences are minimized (Oerlemans & Peeters, 2009). Accordingly, and with respect to intergroup relations, several studies reported greater prejudice towards minority group members when assimilation is endorsed by majority members (Badea, 2012; Levin et al., 2012; Verkuyten, 2005; Wolsko, Park & Judd, 2002). Moreover, when minority group members adhere to assimilation, they are more likely to display negative in-group evaluation (Badea et al., 2015; Verkuyten, 2005).

In short, multiculturalism and assimilation can be distinguished from each other by the way in which equality and social cohesion are achieved, that is by reducing differences (i.e., assimilation) or by the recognition and valorization of those differences (i.e., multiculturalism). Research in the area of immigration and intergroup relationships shows different preferences for multiculturalism and assimilation among members of the majority versus minority groups. In a review of studies on adolescents and young adults in Europe, Verkuyten (2006) reports a constant tendency of minority group members to support multiculturalism. The recent study conducted by Steffens and colleagues (2017) supports the preference of minority participants for a multicultural orientation compared to other ideologies. Moreover, research highlights that majority members do not feel targeted by a multicultural approach, which tends to be perceived as being “about” ethnic minorities (Plaut, Garnett, Bufferdi & Sanchez-Burks, 2011). Moreover, there is evidence in the literature showing that majority group members feel threatened in their national identity within a multiculturalism approach (Ginges & Cairns, 2000; Verkuyten, 2009). Majority group members, thus, are frequently found to favor an assimilation approach (e.g., Dovidio et al., 2007).

The endorsement of multiculturalism and assimilation at the organizational level is much less investigated than at the individual level (Courtois et al., 2014). We can define these perspectives within the organizational context as general values regarding how groups should include and accommodate one another and how to best organize a diverse organization (Cox & Blake, 1991; Ely & Thomas, 2001). Even in the organizational context, minority groups have been generally found to benefit more from multiculturalism than majority groups (Arasaratnam, 2013; Plaut, Thomas, & Goren, 2009). For example, McKay and colleagues (2007) found that all employees felt more committed to their organization when they perceived their organization as willing to support a multicultural approach, but that Black employees (i.e., minority group) showed an additional benefit in terms of reduced turnover intentions in comparison with White and Hispanic subgroups. A study conducted in the context of nursing (Beheri, 2009) showed nurses to be more satisfied in their job when valuing differences with other culturally diverse groups and when having high trust levels with other culturally diverse groups, demonstrating a clear association between job satisfaction and the degree to which cultural differences are valued. Compared to the multiculturalist approach, few studies have investigated effects of the organizational assimilationist perspective of diversity on work outcomes. As an example, Olsen and Martins’ (2016) experimental study showed that multiculturalism and assimilation have similar and better effects than no diversity management policy regarding organizational attractiveness in minority and majority students. They did not find that the effects of the manipulated acculturation strategies on organizational attractiveness were moderated by racioethnic group membership.

### Dual identity

Up to now, dual identity has been investigated in different areas of psychological research, such as intergroup relations and acculturation studies, making it difficult to reach consensus about its definition and measurement (Fleischmann & Verkuyten, 2016). Nevertheless, results on dual identification with one’s ethnic and national identity report many benefits for migrants, such as a better integration process, less stress and better intergroup relations (e.g., Berry, 1997; Nguyen & Benet-Martinez, 2013; Dovidio, Gaertner & Saguy, 2007). Dual identity leads to positive outcome because it prevents group identity from being threatened by ensuring minorities’ need for distinctiveness while maintaining the beneficial effects of a common identity (Dovidio et al., 2007). In the workplace, minority and majority members can be made aware that they are part of different social groups: they are simultaneously member of a cultural group (i.e., group identity), and member of a working group (i.e., organizational identity). The combination of the two is what we refer here with the term dual identity: a superordinate identity allowing people to recognize and endorse distinct subgroup identities, while also convey shared visions and values. Members of minority groups prefer to acknowledge group-based differences along with commonality (Saguy, Dovidio, & Pratto, 2008), that is dual identity, and policies such as multiculturalism that support ethnic differences within a larger society (Dovidio, Gaertner, & Saguy, 2007; Ryan, Hunt, Weible, Peterson, & Casas, 2007; Verkuyten, 2006). Minorities prefer dual identity mainly because focusing only on common identity is perceived as an ethnic identity threat (Crisp, Stone, & Hall, 2006). However, few empirical evidences can be found in the literature accounting for the study of dual identity and its antecedences and consequences. More is found on studies accounting separately for group identity and organizational identity. With respect to antecedents, in their study, Lujiters and colleagues (2006) showed the importance of cultural empathy (i.e., the interest in the outgroup culture) for reaching positive judgments toward a minority target under cultural maintenance conditions. Thus, multiculturalism rather than assimilation may lead to more positive effects in minorities in terms of group identification. In this vein, the study of Badea (2012) reported that majority group’s national identification was positively related to assimilation and negatively to multiculturalism. Verkuyten and Yildiz (2007) demonstrated in their experimental study that people’s views on minority rights depend on group status. More specifically, the authors shown that when people are in a majority status condition, they display a reduced endorsement in favor of minority rights compared to when they are in a minority status condition. Minority rights are thus typically seen as having more to offer to minority groups than to majority groups, whose members may feel cultural diversity as a threat to their dominant position. In terms of organizational identity, Verkuyten and Yildiz (2007) report minority workers to prefer strong team identity adoption (i.e., common identity in their study) to weak team identity adoption.

With respect to assimilation, the process of depersonalization may contribute the identification to the common identity because it is socially structured, in the sense of being shaped by, and oriented towards, the emergent norms of the group as a whole (Haslam, Postmes, & Ellemers, 2003). In general, members of majorities groups show preferences for common identity and policies that promote it, i.e. assimilation (Verkuyten, 2006). Supporting a common identity reduces tensions and produces positive intergroup attitudes and at the same time reinforces social values that maintain the status quo in favor of the majority groups (Dovidio, Gaertner, & Saguy, 2007). Members of majorities groups show preferences for common identity (Verkuyten, 2006); on the other hand, majority groups may not perceive dual identity as such a threat, because it conveys shared values with the mainstream culture. In terms of outcomes and consequences, most studies so far focused on psychological adjustment and quality of life rather than work-related outcomes. For example, Utsey, Chae, Brown and Kelly (2002) showed ethnic identity to be positively related to quality of life in three minority groups (African American, Asian and Latino American participants). Another study by Fingerhut, Peplau, and Ghavami (2005) reported a link between both group identity and common identity with life satisfaction among lesbians in the United States. These studies show that dual identity has positive effects with respect of psychological adjustments and intergroup relationships. In terms of work outcomes, a study conducted Iweins and colleagues (2013) has shown that the more young workers developed a dual identity combining an age-group identity and an organizational-superordinate identity, the less they were prejudiced against older workers and the less they were willing to quit the job. Moreover, positive effects of the multi-age organizational climate on prejudice reduction and intentions to quit were mediated by dual identity. Finally, the ability of migrants to develop a dual identity has been shown to depend on majority’s acceptance of minorities (Bourhius, Moïse, Perreault, & Senecal, 1997; Fleischmann & Phalet, 2015), so that the multicultural approach is generally more beneficial for minority members’ wellbeing than assimilation, separation and marginalization strategies (Berry et al., 2006; Nguyen & Benet-Martinez, 2013). We assume that the same might be true in the workplace.

### Hypotheses

**Figure 1:**
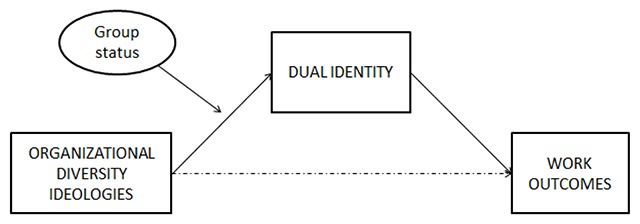
Theoretical model.

Following the results of studies displaying positive advantages of dual identity and acculturation studies showing differences in preferences among minority and majority members, we aim to test a moderated mediation model (shown in Figure 1) in which the indirect effects of acculturation approaches (i.e., multiculturalism and assimilation) on work-related outcomes (i.e., job satisfaction and intention to quit) through the identification of workers with both the organization and their ethnic groups simultaneously (i.e., dual identity) are moderated by the belongingness of participants to either the majority or the minority group. Literature shows that multiculturalism may favor shared cultural attitudes such as support for diversity policies in both minority and majority (Wolsko et al., 2006). However, multiculturalism has been shown to positively contribute to minority group members’ identity (Verkuyten et al., 2005), while majority group members are less prone to support multiculturalism which is perceived as excluding them in general (Plaut et al., 2011). Assimilation, in contrast, ignores differences and instead focuses on a sense of shared humanity (Park & Judd, 2005). An organization adopting an assimilation perspective will ignore ethnic differences among its employees and will absorb them into the dominant culture. In the context of the present study as well as in many Western organizations, the dominant culture is often based on the culture of the majority group (i.e., the White majority, Olsen & Martins, 2016). Thus, majority group members are more willing to endorse an assimilation perspective (see Hehman, Gaertner et al., 2012) and their group self-esteem benefits more from assimilation than minority (Wolsko et al., 2006).

Based on the above, we assume that:

Hypothesis 1. Indirect effects of organizational multiculturalism on work-related outcomes through dual identity are stronger for the minority group than for the majority group of workers.

Hypothesis 2. Indirect effects of organizational assimilation on work-related outcomes through dual identity are stronger for the majority group than for the minority group of workers.

## Methods

### Participants and Procedure

Data were collected online by means of a French-language questionnaire and using a snowball sampling procedure. Of the 609 people who started to fill in the survey, 261 (42.9%)^2^ fully completed it and were included in the study. Of these, 184 participants (70.6%) were of Belgian origin (majority group). 77 (29.4%) had another origin than one of the 27 States members of the European Union; they were the minority group.^3^ More specifically, the vast majority of participants of the minority group (66.2%) were of Moroccan origin or another country of North Africa (15.6%). The average age of participants was almost 38 years old (*M* = 37.66, *SD* = 10.45). Regarding gender, 168 employees (64.4%) were female. The vast majority of the sample held a high school degree (70.5%), worked as a permanent employee (74.3%) and on a full-time basis (72%). Participants were employed in a broad array of sectors, including public administration (23.4%), public health (5%), education (8.8%) and industry (3.8%).

### Measures

#### Organizational assimilation

Organizational assimilation was measured using 8 items adapted from Wolsko and colleagues (2006) (e.g., “For my organization, foreign people should adopt the working habits of Belgian people”). Items were scored on a 7-point asymmetrical rating scale ranging from 1 (completely disagree) to 7 (completely agree). The alpha coefficient of reliability (α) was .87.

#### Organizational multiculturalism

Organizational multiculturalism was measured using 6 items adapted from Wolsko and colleagues (2006) (e.g., “For my organization, it is important that employees with a different origin have the right to maintain their own cultural traditions at work”). Items were scored on a 7-point rating scale ranging from 1 (completely disagree) to 7 (completely agree). The coefficient α was .89.

#### Origin

Origin was coded as a dichotomous variable with two possible values (1 = Belgian origin, majority group; and –1 = foreign origin, minority group).

#### Dual identity

Dual identity was measured by multiplying respondents’ group identity (ingroup) with their organizational (common) identity (for a similar procedure, see Hofhuis et al., 2012; Iweins et al., 2013). Group identity was measured with four items (adapted from Doosje et al., 1995) (e.g., “I identify with the group of Belgian workers”). Organizational identity was measured with the same four items adapted to the organizational identity (e.g., “I see myself as a typical member of my organization”). Because participants responded to all items using a scale ranging from 1 (totally disagree) to 7 (totally agree), participants’ dual identity scores (group identity*organizational identity) ranged from 1 (lowest possible average score on both components) to 49 (highest possible average score on both components). Overall, the alpha coefficients were .93 for the group identity and .90 for the organizational identity.

#### Job satisfaction

Job satisfaction was measured with four items scale of Eisenberger and colleagues (1997) (e.g., “All in all, I am very satisfied with my current job”). Items were scored on a 7-point rating scale ranging from 1 (completely disagree) to 7 (completely agree). The coefficient α was .92.

#### Intention to quit

Intention to quit was measured with four items designed to measure the strength of the participant’s intention to leave its current position (Price, 1997) (e.g., “If I could, I would quit today”). Items were scored on a 7-point rating scale ranging from 1 (completely disagree) to 7 (completely agree). The coefficient α was .92.

#### Control variables

Participants were required to indicate their age (in number of years), gender (female vs male), and level of education. Age and level of education were found to be significantly linked to the mediator (i.e., dual identity), gender to the dependent variable ‘Intention to quit’ and level of education to the dependent variable ‘Job satisfaction’. Accordingly, these variables were introduced as control variables in the subsequent analyses (Becker, 2005). More precisely, all analyses presented later were conducted twice, i.e. with and without control variables. The results were essentially identical across the analyses. The findings discussed below are based on the analyses that include the control variables.

### Data analyses

First, we performed confirmatory factor analyses (CFA) to examine the distinctiveness of our five constructs (i.e., organizational assimilation, organizational multiculturalism, dual identity, job satisfaction, intention to quit). Subsequently, we performed descriptive statistics (means and SDs) and correlations among our variables. Then we analyzed the relationships between diversity models (i.e., organizational assimilation and organizational multiculturalism), dual identity and work-related outcomes, as well as potential differences on these relations based on origin, by performing Multi-group Structural Equation Modelling. Finally, we computed bootstrap analyses to assess the conditional indirect effects (Preacher & Hayes, 2004, 2008). Descriptive and bootstrap analyses were performed using SPSS for Windows, release 21. Confirmatory factor analyses and Multi-group Structural Equation Modelling analyses were conducted using LISREL 8.80 (Jöreskog & Sörbom, 2006).

## Results

### Confirmatory factor analyses

Confirmatory factor analyses (CFA) results were evaluated using the χ^2^ statistic, including its normed version (Jöreskog, 1969), and a variety of other fit indices (i.e., the root mean square error of approximation (RMSEA), the non-normed fit index (NNFI), and the comparative fit index (CFI). As suggested by Schweizer (2010), values for the RMSEA lower than .08 were considered acceptable; values for the NNFI and CFI equal to or higher than .90 were considered acceptable, while values close to .95 or higher were considered good. We compared the fit of the five-factor model with that of a series of more constrained and nested models and made the comparison using χ^2^ differences tests (Bentler, 1990). As expected, the model specifying five latent constructs was clearly superior in terms of fit to all the other models tested, which were not acceptable. The hypothesized five-factor model was thus retained as the best depiction of data. All the items loaded reliably on their predicted factors, with standardized loadings ranging from .48 to .94. Table 1 reports the results for all the measurements models that were tested.

**Table 1:** Goodness-of-fit indices for confirmatory factor analysis.

Model	*χ*^2^	Df	*χ*^2^/df	RMSEA	NNFI	CFI

5-factor model	744.75	289	704.65	.07	.94	.94
4-factor model (MC and AS = 1 factor)	1562.48	293	2263.87	.16	.83	.84
4-factor model (JS and IQ = 1 factor)	960.34	293	1011.89	.10	.91	.92
4-factor model (DI and JS = 1 factor)	1678.69	293	2010.12	.15	.81	.83
4-factor model (AS and DI = 1 factor)	1597.73	293	1664.21	.13	.82	.84
3-factor model (MC and AS = 1 factor; JS and IQ = 1 factor)	1773.99	296	2597.53	.17	.80	.82
3-factor model (DI, JS and IQ = 1 factor)	2454.57	296	4002.27	.22	.71	.74
3-factor model (AS and DI = 1 factor; JS and IQ = 1 factor)	1813.27	296	1968.97	.15	.80	.81
3-factor model (MC and AS = 1 factor; DI and JS = 1 factor)	2792.02	296	3579.17	.21	.70	.73
2-factor model (MC and AS = 1 factor; DI, JS and IQ = 1 factor)	3268.22	298	5542.89	.26	.60	.64
2-factor model (MC, AS, and DI = 1 factor; JS and IQ = 1 factor)	2625.69	298	3675.02	.21	.69	.72
1-factor model	3467.03	299	5096.81	.25	.58	.61

*Note*: *N* = 261. χ^2^ = Chi-square Test; χ^2^/df = Normed Chi-square; RMSEA = Root mean square error of approximation; NNFI = Non-normed fit index; CFI = Comparative fit index; MC = Organizational multiculturalism; AS = Organizational assimilation; DI = Dual identity; JS = Job satisfaction; IQ = Intention to quit.

### Preliminary analyses

Means, SDs, and correlations regarding the study variables are displayed in Table 2. Organizational diversity models (i.e. assimilation and multiculturalism) were positively related to the mediator, i.e. dual identity (majority group, *r* = .21, *p* < .01 for assimilation; minority group, *r* = .50, *p* < .001 for multiculturalism). Results of correlations analyses showed organizational multiculturalism to be significantly related to the dependent variables, namely job satisfaction (*r* = .39, *p* < .01) and intention to quit (*r* = –.35, *p* < .01) in the minority group. Organizational assimilation was found to be marginally linked to job satisfaction (*r* = .13, *p* = .08) in the majority group. Dual identity was also found to be positively related to job satisfaction (*r* = .36, *p* < .01) and negatively related to intention to quit (*r* = –.34, *p* < .01).

**Table 2:** Means, Standard Deviations, and Correlations between variables.

	*M*	*SD*	1	2	3	4	5	6	7	8

0. Origin	–	–	–	–.07	.17**	.22***	.15**	.01	.11^†^	–.02	.02
1. Gender	–	–	–		–.10	–.15*	.00	.08	–.13^†^	.22**	.11
2. Age	37.66	10.45	–	.36**		.02	.12	–.08	.02	–.04	–.16*
3. Level of education	–	–	–	–.16	–.24*		–.19*	.13^†^	.19*	–.10	–.11
4. Organizational assimilation	5.62	.95	.87	.10	.05	–.10		–.35**	.13^†^	–.05	.21**
5. Organizational multiculturalism	4.31	1.36	.89	.09	.01	–.19^†^	–.06		.06	–.04	.06
6. Job satisfaction	5.30	1.36	.92	–.02	.04	.01	.06	.39***		–.78***	.16*
7. Intention to quit	2.42	1.52	.92	–.08	–.19	.07	–.02	–.35**	–.74***		–.16*
8. Dual identity	20.53	10.99	(.93)^a^ (.90)^b^	.01	–.03	–.19	.16	.50***	.36**	–.34**	

*Note*: Mean, Standard Deviation and Reliability estimates (α) calculated on the total sample (*N* = 261). Origin was coded 1 = Belgian and –1 other. Gender was coded 1 = males and –1 females. Level of education was coded 1 = low educated, 2 = medium educated and 3 = high educated. ^a^ Cronbach’s alpha for Group Identity; ^b^ Cronbach’s alpha for Organizational Identity. Coefficients of the majority group (N = 184) appear above the diagonal and of the minority group (N = 77) appear below the diagonal. * *p* < .05; ** *p* < .01; *** *p* < .001, ^†^ p < .10.

### Tests of hypotheses

First, we assessed across our two sub-groups (i.e., majority and minority groups) the hypothesized structural relationships among latent variables, which propose a full mediation between diversity models and work-outcomes through dual identity. The hypothesized model adequately explained the data, χ^2^(793) = 1904.25 (χ^2^/df = 2.40; RMSEA = .10; CFI = .90; NNFI = .89). We then compared the model with four alternative models, each of them containing additional paths that suggest partial rather than full mediation. We added, separately, direct links between organizational multiculturalism and job satisfaction, between organizational multiculturalism and intention to quit, between organizational assimilation and job satisfaction, and between organizational assimilation and intention to quit (Models 2, 3, 4, and 5, respectively). Fit indices for each of these alternative models are presented in Table 3. The χ^2^ difference test indicated Model 2 as the best model to depict the data, but examination of the parameters indicated a non-significant link between organizational multiculturalism and job satisfaction (γ = .07, *p* > .05). We thus considered Model 1 (namely, the theoretical model) as the best one to represent our data. For the sake of clarity, the effects of the control variables are described in the text. Age was found to be significantly related to dual identity (γ = –.15, *p* < .01), while level of education and gender did not predict significantly any of the considered variables (i.e., dual identity, job satisfaction and intention to quit). Results showed that organizational multiculturalism and organizational assimilation are positively associated with dual identity (γ = .27, *p* < .001 and γ = .27, *p* < .001, respectively), dual identity is positively related with job satisfaction (β = .28, *p* < .01) and negatively associated with the intention to quit (β = –.26, *p* < .01).

**Table 3:** Fit indices for Multigroup SEM analyses.

Model	*χ*^2^	Df	Δ *χ*^2^ (Δ df)	*χ*^2^/df	RMSEA	CFI	NNFI	Model comparison

Model 1 (hypothesized)	1904.25	793	–	2.40	.10	.90	.89	
Model 2: adds path between MC and JS	1897.52	792	6.73 (1)	2.39	.10	.90	.89	Model 1 vs Model 2
Model 3: adds path between MC and IQ	1894.93	791	2.59 (1)	2.39	.10	.90	.89	Model 2 vs Model 3
Model 4: adds path between AS and JS	1896.71	791	0.81 (1)	2.40	.10	.90	.89	Model 2 vs Model 4
Model 5: adds path between AS and IQ	1897.16	791	0.36 (1)	2.40	.10	.90	.89	Model 2 vs Model 5

*Note*: N = 261. MC = organizational multiculturalism; AS = organizational assimilation; JS = job satisfaction; IQ = intention to quit; χ^2^ = Chi-square Test; df = degree of freedom; Δ χ^2^ = Chi^2^ difference tests between the best fitting model and alternative models; χ^2^/df = Chi^2^ goodness of fit to degrees of freedom ratio; RMSEA = Root Mean Square Error of Approximation; CFI = comparative fit index; NNFI = Non-normed fit index.

Then, we conducted a series of multi-group analyses to test the moderating effect of origin (minority vs majority) on the mediation between organizational diversity ideologies and work-related outcomes through dual identity. Employees of Belgian origin (i.e., majority group) (*N* = 184) formed the first group, while employees of foreign origin (i.e., minority group) (*N* = 77) formed the second group. The baseline model for comparison purposes was the hypothesized model retained earlier (Model 1 in Table 4), that is a completely invariant model in which all measurement and structural parameters were constrained to equality across sub-group (i.e., a model where minority and majority groups are equivalent – no effect of origin on our structural path of interest). We then relaxed the equality constraint across sub-groups for each structural parameter at a time (i.e., in order to test whether the strength of the path differs from one sub-group to the other, suggesting a moderating effect of the origin). The models were compared using a χ^2^ difference test. Results reported in Table 4 show that the model where the path between organizational multiculturalism and dual identity was freely estimated in each group displays a significant increase in model fit compared with the baseline model (χ^2^ difference = 12.81, df = 3, *p* < .05). On the contrary, freely estimating the path between organizational assimilation and dual identity across the group (χ^2^ difference = –.25, df = 1, *ns*) does not improve the fit.

**Table 4:** Results of Multigroup analyses.

Model	*χ*^2^	Df	CFI	NNFI	RMSEA	Δ *χ*^2^	*p*

Model 1 (groups equivalent)	1904.25	793	.90	.89	.10		
Model 2 (groups different on the path MC-DI)	1891.44	790	.90	.89	.10	12.81	<.05
Model 3 (groups different on the path MC-AS-DI)	1891.69	789	.90	.89	.10	–0.25	ns

*Note*: N = 261. MC = organizational multiculturalism; DI = dual identity; AS = organizational assimilation; χ^2^ = Chi-square Test; df = degree of freedom; GFI = Goodness of Fit Index; NFI = Normed Fit Index; NNFI = Non-normed fit index; RMSEA = Root Mean Square Error of Approximation. These results suggest a moderating effect of origin on the relationship between organizational multiculturalism and dual identity, with a positive and significantly stronger path between organizational multiculturalism and dual identity for the minority group (β = .49, *p* < .001) compared with the majority group (β = .16, *p* < .05) (see Figure 2). Contrary to our expectations, the effects of organizational assimilation are not moderated by origin since paths do not significantly differ from majority to minority participants.

**Figure 2:**
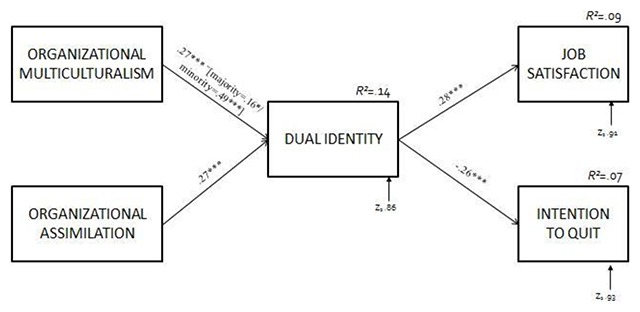
Standardized Path Coefficients for the model identified.

Second, we used Hayes’s (2013) PROCESS macro (model 7) to obtain bias-corrected bootstrapped confidence intervals for the conditional indirect effects (Edwards and Lambert, 2007; Preacher et al., 2007) (results in Table 5). Importantly, the estimates and bias-corrected bootstrapped 95% confidence intervals for the conditional indirect effects using 5000 bootstrap samples indicate that the indirect effect of organizational multiculturalism on both job satisfaction and intention to quit via dual identity is significant for the minority group of workers only (indirect effect =.10; SE = .04; BCa95%CI = [.03; .19]; effect size *R^2^* = .09; indirect effect = –.10; SE = .05; BCa95%CI = [–.21; –.03]; effect size *R^2^* = .07, respectively). The index of moderated mediation (Hayes, 2015) is significantly different from 0 for both analyses (index = –.09; SE = .04; BCa95%CI = [–.18; –.02] for job satisfaction; index = .09; SE = .05; BCa95%CI = [.02; .21] for intention to quit). Finally, results of Hayes’s (2013) PROCESS macro (model 4) revealed that the indirect effect of organizational assimilation on both job satisfaction and intention to quit via dual identity is significant (indirect effect = .06; SE = .03; BCa95%CI = [.02; .13]; effect size *R^2^* = .08, for job satisfaction; indirect effect = –.10; SE = .03; BCa95%CI = [–.14; –.02]; effect size *R^2^* = .05, for intention to quit).

**Table 5:** Conditional Indirect Effects Multiculturalism on work outcomes.

Level of moderator	Indirect effect	Boot SE	Boot LL CI	Boot UL CI

	Multiculturalism ➔ Dual Identity ➔ Satisfaction			
Minority group	.10	.04	.03	.19
Majority group	.01	.02	–.02	.05
	Multiculturalism ➔ Dual Identity ➔ Intention to quit			
Minority group	–.10	.05	–.21	–.03
Majority group	–.01	.02	–.06	.02

*Note*: Number of bootstrap samples for bias corrected bootstrap confidence intervals: 5,000. Level of confidence for all confidence intervals: 95 per cent. SE = standard error, LL = lower level, CI = confidence interval, UL = upper level, SD = standard deviation.

## Discussion

Our study was conducted to test the general hypothesis that the effects of multiculturalism and assimilation (as endorsed at the organizational level) on job satisfaction and intentions to quit are mediated by dual identity and moderated by origin (minority vs majority groups). In particular, we assumed stronger indirect effects of organizational multiculturalism for the minority group, and stronger indirect effects of organizational assimilation for the majority group of workers. We collected data in Belgium, where studies on diversity are still sparse in the field of organizational behavior, by means of a quantitative survey. Our multi-group analysis nicely complements research on diversity management that is mainly qualitative (Al Ariss & Crowley-Henry, 2013). Moreover, our study answers to the criticism that diversity management is primarily supportive for the position of minority groups (Ashikali & Groenveld, 2015) by analyzing diversity from the point of view of minority and majority groups. Finally, our findings provide empirical evidence that help to better understand key processes that still remain little understood in ethnic minorities’ careers (Al Ariss & Crowley-Henry, 2013; Akkermans & Kubbasch).

First of all, our findings show that both organizational multiculturalism and assimilation are beneficial diversity perspectives for all workers. In other words, congruently with Olsen and Martins (2016) who showed that multiculturalism and assimilation were more related to organizational attractiveness than no-diversity context, we found that the more the workers perceived that their organization considered diversity (either by valuing or by denying differences between groups), the higher was their subjective higher job satisfaction, and the lower was their intention to quit.

In line with the literature that posits multiculturalism to be more beneficial for minorities (Rosenthal & Levy, 2010), our study shows that organizational multiculturalism holds stronger positive effects for the minority group of workers than for the majority group. We showed that dual identity mediates the effects of organizational multiculturalism on work-related outcomes for both groups of workers considered, but particularly for members of the minority group. By doing so, our findings complement the few previous studies that have found that dual identity patterns are likely to emerge within the context of multiculturalism (e.g., Berry, 2001; Hofhuis et al., 2012; Iweins et al., 2013).

Multiculturalism has been sometimes criticized in reason of the risk for minority group members to not engage in the host society and retreat in themselves (e.g., Igarashi, 2019). However, our study demonstrated that a multicultural approach is beneficial in terms of work outcomes for minority group members. This may be explained by the fact that, in contexts in which tolerant multicultural policies are endorsed, the cultural distance between the national identification of natives and immigrants is small.

Our assumption that organizational assimilation would generally have the most beneficial effects for the majority group of workers was not confirmed. Indeed, origin did not moderate the relationship between organizational assimilation and dual identity. We found that the positive versus negative relationships between organizational assimilation and both job satisfaction and intention to quit, respectively, are mediated by dual identity, regardless of the group (i.e., majority vs minority) of workers. Our findings suggest that creating a positive climate of equality of treatment has positive effects for all workers, including minority workers. It is in fact the major point of assimilation to promote the idea of individuals being all the same, regardless of origin. Individuals from ethnic minorities may be more concerned about culture maintenance in the private domain of life, rather than in the public domain (e.g., workplaces), where they might prefer to assimilate the dominant culture and habits of the majority group (Arends-Tóth & van der Vijver, 2004; Peeters & Oerlemans, 2009). Moreover, ethnic minority might benefit from assimilation due to reduced cultural distance (Triandis, 1994, 2000). Cultural distance is less salient in contexts which share the same languages, social structures, religions, standards of living, and values (Triandis, 2003, p. 489).

The key factor of the positive effects of organizational multiculturalism and organizational assimilation is the dual identity, that acknowledges and values both similarities (i.e., organizational identity) and differences (i.e., group identity). In both groups, stronger dual identity predicted higher subjective career success (i.e., job satisfaction) as well as a more likely stable career (i.e., lower turnover intentions).

Finally, our study provides empirical evidence that management of diversity and dual identity contribute to subjective success in the career (i.e., one’s positive interpretation of one’s career situation, Tharmaseelan, Keer, & Carr, 2010: 220). For example, Tharmaseelan et al. (2010) found that the adaptation to the single host culture was not enough to predict subjective career. Our study points out the importance of the combination between group and organizational identities (i.e., dual identity), especially in a multicultural context for the minority group. The more the workers perceived that their organization considered diversity, the more they felt their situation as successful, that is, the higher was their current job satisfaction, and the more likely was their future integration in the organization (i.e., lower intention to quit).

### Limitations and research perspectives

Our study has some limitations that should be taken into account. First of all, because of its cross-sectional design, we are not able to conclude on the causality of the relationships we suggested. However, we tested reverse causality (analyses are available on demand). Findings show that a model where assimilation and multiculturalism predict work-related outcomes through dual identity is significantly better than a model where dual identity would predict work-related outcomes through assimilation and multiculturalism. Therefore, on the basis of both theoretical and empirical evidence, we are confident that our model is the best one to explain our findings.

Second, the size of our sample, especially the minority group subsample, may represent a limitation of our statistical analyses. However, we should note that origin-based minority groups are often represented through smaller samples in studies. The rate of our minority group subsample (29.4% of the total sample) is similar to – or even better than – other samples (for example 21.7% in Mckay et al., 2007; 21% in Plaut et al., 2009).

Thirdly, we did not control for the possible social desirability in participants’ responses, their political orientation as well as the relationship between ethnic background, i.e. elements that may have an influence on integration preferences displayed by minority and majority groups. Therefore, future work on diversity in the workplace should take into account the limitations of the present study. Another important limitation of our study is that we did not measure cultural distance (Triandis, 1994), that is we did not control for how cultural distant from the natives (i.e., majority group) are the members of our minority group.

Lastly, our study accounts for only assimilation and multiculturalism, while other acculturation strategies exist, such as republicanism, that is the French form of colorblindness (i.e., a political system where collective identity is created by a voluntary membership of the citizens (and not communities) in shared principles and values (Kamiejski, Guimond, De Oliveira, Er-Rafiy, Brauer, 2012, 53). Republicanism has been shown to correlate to both assimilation and multiculturalism (Badea, 2012), thus it would have been of interest to investigate this approach in the Belgian context where both assimilation and multiculturalism are present and promoted at the same time.

Our research allows us to provide insights and directions for future research. As recently suggested by Guillaume and colleagues (2014), studies on diversity management strategies are needed in order to promote the well-being of different groups in modern organizations. In this respect, we believe that our study is an important contribution in the direction of learning more about the role of the assimilation and multiculturalism at the organizational, rather than the individual, level. Results of our study report, to some extent, benefits in both groups of workers from both multiculturalism and assimilation. The positive relationships found in the present study between assimilation and work-related outcomes can be viewed in line with the literature that proposes to re-consider assimilation as an alternative to multiculturalism and to rehabilitate this diversity model which has been subject of criticism in the past decades (Alba & Nee, 1997; Gans, 1999).

In relation with dual identity, our findings reveal that, just as members of the minority group do not seem to feel excluded from assimilation, members of the majority group do not seem to feel excluded from multiculturalism, even though effects were less positive for them than for members from the minority group. Future research should explore other possible routes that could explain such positive effects of assimilation and multiculturalism. In this vein, feeling of inclusion, that is the perception to belong to the group as a distinct and acknowledged member of this group (Shore et al., 2011), offers a quite promising perspective. Additionally, beyond origin, other boundary conditions of the positive effects of multiculturalism and assimilation should be identified. Olsen and Martins (2016) showed that being a few minority group members in the majority group (rather than more balanced groups) induced more positive effects of multiculturalism compared with assimilation. Consequently, future research should better take into account the characteristics of the objective (i.e., numeric) diversity of the context in the analysis of diversity models effects.

Finally, our study did not take into account that individuals may be simultaneously integrated in the mainstream society (and working context) while prejudice and discrimination can still be widespread (Badea, 2012; Levin et al., 2012). Even though assimilation has been shown to lead to positive outcomes in the minority group of our study, we have to keep in mind that a working context that does not take into account differences among workers might be perceived as providing injustice and disrespectful treatment, especially in members of highly stigmatized groups (such as minority groups). Previous studies have shown that multicultural support to diversity from the organization can buffer effects of discrimination among workers (e.g., Triana et al., 2009). Future research should examine whether and how assimilation can play a similar protecting role.

### Managerial implications

In recent years, the flow of migrant workers from various part of the world toward western – especially European – countries has significantly increased and has major consequences for labor markets (Akkermans, & Kubbasch, 2017). Despite these demographic trends, ethnic minorities are still under-represented in the workforce, and organizations have to improve their diversity management strategies for moral and legal requirements as well as in order to attract and retain the best human resources and to ensure their efficiency (Cox, 1993; Olsen & Martins, 2016; Yang & Konrad, 2011). Our study shows that there are potentially several routes to reach this objective, i.e. multiculturalism and assimilation (see also Olsen & Martins, 2016).

In their review of the literature, Yang and Konrad (2011) underlined that managers are likely to adopt diversity management practices directed either toward multiculturalism or to assimilation because they have various views about the value of diversity for the creation and implementation of effective business strategies. On the basis of our study that shows positive effects of both multiculturalism and assimilation, organizations should encourage their managers to develop a complex approach of diversity that acknowledges the two diversity perspectives to take advantages of each. On the one hand, organizations should pay attention to provide conditions of fairness in diversity to be inclusive of all groups, for example in hiring or promotion procedures. Organizational assimilation is well suited for this. Our study showed positive effects for all workers. On the other hand, organizations should not deny ethnic differences because multiculturalism seems of prime importance for minorities, in a way that it could help them to enhance identity processes, facilitating their well-being and intention to stay at work. Ethnic differences might be acknowledged in team collaboration where mutual cultural learning among workers could be supported by managers and valued by the organization (Ely & Thomas, 2001). It should be noted that, despite both diversity models have positive effects, promoting organizational multiculturalism might be conceived as a better choice in terms of well-being at work of minority workers.

Dual identity has been shown to convey positive effects of workers’ perceived organizational multiculturalism and assimilation. Therefore, our findings show that organizations should develop a “bi-cultural competency” in their workers (La Fromboise et al., 1993, in Bell & Harrison, 1996) that combines a common background (a supra-ordinate identity like the organizational identity) and the acknowledgment of differences (group or cultural identity) when they implement diversity policies and practices. This “bi-cultural competency” (i.e., dual identity) contributes higher levels of job satisfaction and lower intentions to quit in minority as well as majority workers. This result is of particular interest organizations that are more and more concerned with diversity.

## Conclusion

This study underlines that both assimilation and multiculturalism, as endorsed at the organizational level, positively contribute to work-related outcomes for majority and minority groups of workers. However, organizational multiculturalism and dual identity hold generally the most beneficial outcomes, especially (but not exclusively) for minorities. Finally, our findings highlight the need to take ethnic and identity issues into account when studying work outcomes in culturally diverse organizations.

## Footnotes

Foreign people are non-Belgian born people and/or having at least one of the two parents non-born in Belgium. These data refer to people from 18 to 60 years old (SPF, 2015).

Data were collected via online survey. We deleted from the study sample people who only clicked the link to the survey and quit it early in the process, and the participants who were not workers (selection criteria).

This study has been conducted before Croatia joined the EU. We referred to the Report for employment, CELCR, 2012) for distinguishing between minority and majority groups on the basis of the membership to the EU-27. Participants of Belgian origin were Belgian-born people or having two Belgian-born parents (CELCR, 2012).
